# Torsion of vermiform appendix: case report and review of the literature

**DOI:** 10.1186/s40792-020-0771-x

**Published:** 2020-01-08

**Authors:** Kosuke Endo, Masahito Sato, Kenichi Saga, Atsushi Higashio, Yoshiaki Yuba, Yoshiki Morotomi

**Affiliations:** 1 Department of Pediatric Surgery, Kitano Hospital, The Tazuke Kofukai Medical Research Institute, 2-4-20 Ohgimachi, Kita-Ku, Osaka, 530-8480 Japan; 2 Department of Pathology, Kitano Hospital, The Tazuke Kofukai Medical Research Institute, 2-4-20 Ohgimachi, Kita-Ku, Osaka, 530-8480 Japan

**Keywords:** Torsion, Vermiform appendix, Appendicitis

## Abstract

**Background:**

Torsion of the vermiform appendix is a rare disease with symptoms very similar to those of acute appendicitis. We herein report a case of torsion of the vermiform appendix diagnosed by intraoperative findings.

**Case presentation:**

A 4-year-old boy presented to our hospital because of abdominal pain and vomiting. Laboratory data revealed a C-reactive protein level of 0.08 mg/dL and white blood cell count of 19,300/μL (neutrophils, 88.9%). Abdominal ultrasound showed a target sign-like finding in the ileocecal region. A computed tomography scan showed swelling of the appendix. We performed an emergency operation under suspicion of acute appendicitis. Laparoscopic examination showed that the appendix had twisted 720° in the clockwise direction. Appendectomy was performed, and the postoperative course was uneventful.

**Conclusions:**

Although torsion of the vermiform appendix is a very rare disease and difficult to differentiate from appendicitis, an inappropriate treatment plan could lead to necrosis and perforation of the appendix. It is important to consider this disease as a differential diagnosis in patients with right lower abdominal pain.

## Background

Torsion of the vermiform appendix is a rare disease with symptoms very similar to those of acute appendicitis. Interval appendectomy for acute appendicitis has recently become popular. However, the use of conservative therapy with antibiotics for torsion of the vermiform appendix can cause necrosis of the appendix, leading to perforation of the appendix and peritonitis.

We herein present a case involving a boy who was admitted under the diagnosis of appendicitis and was subsequently found to have appendiceal torsion on laparoscopy.

## Case presentation

A 4-year-old boy presented to the emergency room with abdominal pain. Abdominal ultrasonography (US) revealed no apparent cause of the pain. A massive bowel movement was achieved by an enema and the patient’s pain improved; thus, he was discharged home. However, he developed vomiting and intermittent abdominal pain than night and presented to our hospital again the following day.

**Fig. 1:**
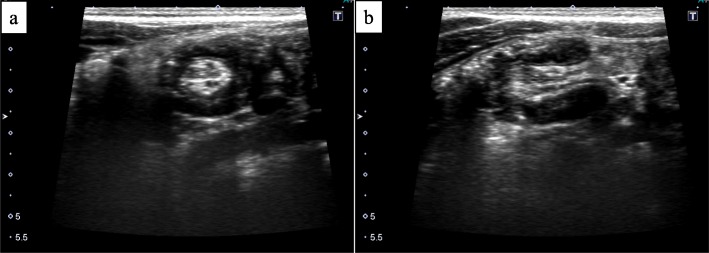
Ultrasonography of the abdomen. **a** Axial view of the base of the appendix showed target sign-like appearance. **b** Longitudinal view showed pseudo-kidney sign

**Fig. 2:**
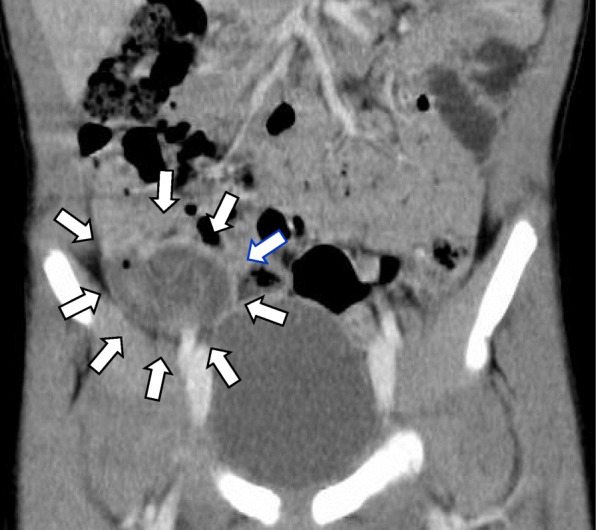
Computed Tomography scan of the abdomen. Swollen appendix (arrow) can be seen

Assessment of the patient’s general appearance indicated that he was very painful, and his body temperature was 37 °C. Physical examination revealed tenderness throughout the entire abdomen with the maximum point in the right lower quadrant. Laboratory data revealed a C-reactive protein level of 0.08 mg/dL and white blood cell count of 19,300/μL (neutrophils, 88.9%). Abdominal US showed target sign-like findings in the ileocecal region (Fig. 1), and these findings were slightly different from the typical findings of intussusception. Enhanced computed tomography (CT) showed a swollen appendix (Fig. 2). An emergency operation was performed under the diagnosis of acute appendicitis.

**Fig. 3:**
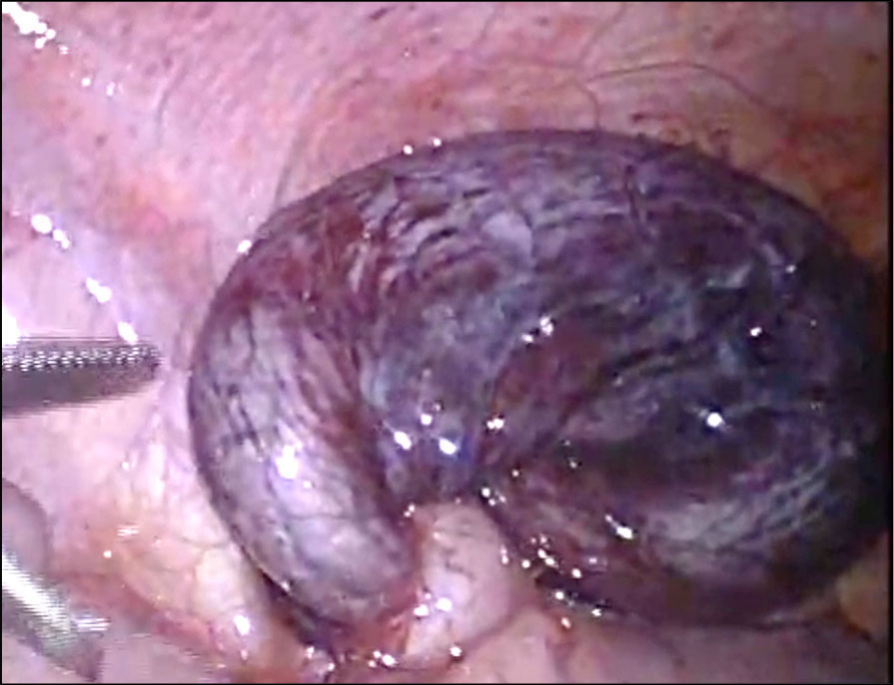
Laparoscopic finding. The appendix was twisted 720° clockwise direction

**Fig. 4:**
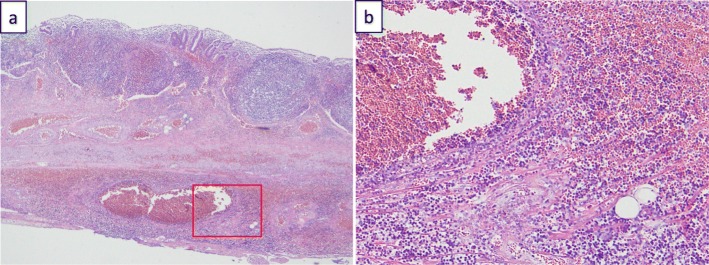
Histopathological findings of the appendix (Hematoxylin & Eosin staining). Mononuclear cell infiltration was observed mainly in serosa rather than mucosa, indicating that the possibility of acute appendicitis is low. There was no evidence of neoplasia. (**a**, original magnification:×40; **b**, original magnification:×400)

The operation was performed using the conventional three-port laparoscopic technique. Laparoscopy showed that the appendix was swollen and dark red in color and that it had twisted 720° in the clockwise direction at its base (Fig. 3). No adhesion was present between the appendix and surrounding tissues. A routine appendectomy was performed. The operation time was 51 min. Histopathological examination revealed an appendix with the main focus of inflammation in the serosa rather than mucosa. There was no evidence of neoplasia (Fig. 4). The postoperative course was uneventful, and the patient was discharged on the fourth postoperative day.

## Discussion

Torsion of the vermiform appendix was first reported by Payne in 1918 [1], and sporadic case reports have since been published. A review of the English-language literature revealed only 22 pediatric cases (including our case), which are summarized in Table 1 [2–19]. The patients’ ages ranged from 11 weeks to 18 years (mean, 7.6 years). The male/female ratio was 18/4. After admission, observation was adopted in two cases and CT-guided drainage was performed in one case. Emergency operations were performed in all three of these patients because the symptoms did not improve in two patients and a dark, foul-smelling bloody aspirate instead of the expected purulent drainage was obtained in the remaining patient.

**Table 1:** Cases of appendiceal torsion reported in the English literature

	Author	Year	Age	Sex	Degree /rotation of torsion	Length, [cm]	Etiology	Preoperative diagnosis	Nausea/vomiting	BT
1	Carter AE	1959	8	F	> 360/CC	-	UD	UD	Vomiting	38.3
2			16	M	> 360/CC	-	UD	UD	Nausea	37.4
3	King-Pan	1965	18	F	1260/C	10	Simple mucocele	Acute appendicitis	No vomiting	37.3
4	Ghent	1966	12	M	360/C	7	Primary	UD	Nausea	37.6
5	Finch	1974	12	M	270/CC	-	UD	UD	Vomiting	37.2
6	Willan	1983	4	M	720/CC	7	UD	Acute appendicits	Vomiting	37.3
7	Dewan	1986	3	M	720/CC	7	UD	UD	Vomiting	37.9
8			6	F	1080/C	7	UD	UD	Vomiting	37.4
9			16	M	−/−	–	UD	UD	UD	Afebrile
10	Waters	1986	3	M	720/−	–	UD	Exploratory lap.	Vomiting	38.9
11	Merret	1992	14	M	720/CC	14	Normal appendix	Acute appendicits	Vomiting	37.5
12	Brian F Gilchrist	1995	6	M	360/CC	9	Long narrow mesoappndix	UD	Vomiting	37.2
13	Val-Bernal JF	1996	6	M	> 360/CC	13.5	Primary	Acute appendicitis	Vomiting	37.5
14	Uroz-Tristan	1998	5	M	360/CC	15	Absent mesoappendix	Torsion or mucocele	No vomiting	No fever
15	Oguzkurt	2004	2	M	270/CC	10	Duplicated colon and appendix	UD	Vomiting	38
16	Gopal K	2005	9	M	720/-	5	Primary	Acute appendectomy	Vomiting	Afebrile
17	Sarin	2006	9	M	270/C	8	Normal appendix	UD	Vomiting	37.7
18	Montes Tapia F	2009	3	M	1080/CC	–	Narrow appendicular mesentery and movable cecum	Acute appendicitis	Vomiting	UD
19	Lena Perger	2011	11w	F	360/CC	–	UD	Acute appendicitis	Emesis	Low-grade fever
20	D’Souza GF	2011	2	M	−/−	6.5	UD	Acute appendicitis	Vomiting	38.3
21	Hirpara DH	2018	2	M	720/C	7.5	Lymphoid hyperplasia	Acute appendicitis or Meckel’s diverticulitis	Emesis	Low-grade fever
22	Our case	2019	4	M	720/C	8	Primary	Acute appendicitis	Vomiting	37.2

Torsion of the vermiform appendix can be primary or secondary. The causes of secondary torsion include mucoceles [3, 20], fecaliths [21], tumors [22, 23], and similar conditions. Possible causes of primary torsion include an abnormal mesentery, such as that with a narrow base, absence of azygotic folds that usually fix the appendix or inflammation; peristaltic bowel movements; the use of laxatives; and vigorous physical exercise. As shown in Table 1, most cases in the pediatric population are primary. In the present case, we found no adhesion between the appendix and the surrounding tissues. Because of the torsion, we could not evaluate the shape of the mesentery. The medical interview revealed that the patient had been playing with a horizontal bar when his symptoms appeared. The enema, which we performed during the first check-up, might have increased the tension within the appendix. Either of these factors could have contributed to the torsion of the vermiform appendix. The pathological examination revealed that the inflammation of the appendix was mainly present in the serosa, not in the mucosal layer, indicating that appendicitis was not the cause of the torsion.

The symptoms of torsion of the vermiform appendix are similar to those of appendicitis. It is difficult to diagnose this entity by preoperative imaging, such as US or CT. In all but one case, the patients were not preoperatively diagnosed with torsion of the appendix. We were also unable to preoperatively diagnose this condition in the present case. In addition to abdominal pain, 17 of 22 patients also developed nausea and vomiting, which may prompt suspicion of other pathogeneses of the abdominal pain. Hamada et al. [20] reported that target-sign like appearance was a useful finding in identifying appendiceal torsion. Although rare, it is important to consider this condition as one of, and if we suspect, the best technique with which to simultaneously diagnose and treat this condition.

The idea of interval appendectomy or nonoperative management for acute appendicitis has recently become popular in the field of pediatrics [24]. The indications for this approach are expanding, and some institutes perform interval appendectomy in both perforated and nonperforated cases. If we adopt this strategy in patients with torsion of the vermiform appendix and treat them with antibiotics, necrosis, and perforation could result. Three cases in which observational treatment was attempted resulted in emergent operation in the end. When we start conservative treatment, we should be aware of the possibility of this rare condition.

## Conclusion

Although torsion of the vermiform appendix is a very rare disease and difficult to differentiate from appendicitis, an inappropriate treatment plan could lead to necrosis and perforation of the appendix. Therefore, it is important to consider this disease as a differential diagnosis in patients with right lower abdominal pain.

## Data Availability

The dataset supporting the conclusions of this article is included in the article.

## Abbreviations

CT: Computed tomography
US: Ultrasonography
